# *Ganoderma lucidum* Polysaccharide Peptide Alleviates Cyclophosphamide-Induced Male Reproductive Injury by Reducing Oxidative Stress and Apoptosis

**DOI:** 10.3390/biomedicines12081632

**Published:** 2024-07-23

**Authors:** Hang Zhang, Nannan Li, Yukun Zhang, Yue Xu, Feng Lu, Dongmei Lin, Shuqian Lin, Min Li, Baoxue Yang

**Affiliations:** 1 State Key Laboratory of Vascular Homeostasis and Remodeling, Department of Pharmacology, School of Basic Medical Sciences, Peking University, Beijing 100191, China; hangzhang@bjmu.edu.cn (H.Z.);; 2 Chongqing Key Laboratory of Development and Utilization of Genuine Medicinal Materials in Three Gorges Reservoir Area, Chongqing 404120, China; 3 Division of Pharmaceutics and Pharmacology, College of Pharmacy, The Ohio State University, Columbus, OH 43210, USA; 4 China National Engineering Research Center on JUNCAO Technology, Fujian Agriculture and Forestry University, Fuzhou 350002, China

**Keywords:** GLPP, chemotherapy, infertility, oxidative stress, spermatogenic stages, apoptosis

## Abstract

Chemotherapy is an important factor leading to male infertility. It is crucial to discover safe and effective treatments to prevent male reproductive injury caused by chemotherapy. The *Ganoderma lucidum* polysaccharide peptide (GLPP) has multiple pharmacological activities. The purpose of this study was to determine whether GLPP could protect the male sperm production from chemotherapeutic injury using a mouse model, with testicular damage induced by cyclophosphamide (CP). CP (50 mg/kg/day) was injected intraperitoneally into male ICR mice gavaged with different doses of GLPP at certain spermatogenic stages. The experimental results showed that GLPP alleviated the CP-induced reduction in reproductive organ coefficients and sperm parameters and reduced the morphological damage of testicular tissues in a dose-dependent manner. GLPP significantly improved the reproductive index, sperm-related parameters, sex hormone levels, and histological testis architecture at different spermatogenic stages. Furthermore, GLPP significantly increased superoxide dismutase (SOD), glutathione (GSH), catalase (CAT), Nrf2, and HO-1, and decreased malondialdehyde (MDA) and Keap-1 in the testicular tissue, indicating reduced oxidative stress. In addition, GLPP limited CP-induced apoptosis via a reduction in Bax expression and increase in Bcl-2 expression. This study suggests that GLPP plays a protective role in spermatogenesis by reducing chemotherapeutic injury and might be developed into drug for male patients receiving chemotherapy.

## 1. Introduction

Infertility has become an important medical problem affecting ~15% of couples worldwide [1,2,3], among which approximately 50% of cases are caused by male infertility [4,5]. Male infertility is caused by multiple factors [6,7,8]. The reproductive toxicity of chemotherapy is considered as a critical factor for male infertility [6,9]. Many chemotherapeutic agents directly exert toxic effects on male germ cells, leading to diminished sperm production, impaired sperm quality, and even infertility [10,11]. There is currently no effective therapeutic drug to treat infertility in patients undergoing chemotherapy. It is necessary to discover new drugs that can protect male germ cells from the damage caused by chemotherapy. Previous studies have shown that some natural products possess promising beneficial effects in reducing chemotherapy-induced toxicity in male germ cells [12,13]. However, there are still some limitations such as the complex extraction process and weak effects.

*Ganoderma lucidum* (GL), a valuable traditional Chinese medicine, has been used for over 2000 years for its medicinal activities [14,15,16,17]. It was recently found that, due to its antioxidant properties, GL has multiple protective effects on tissue injuries and functional damage caused by ischemia-reperfusion injury [18]. In addition, GL can ameliorate age-related changes in structure, morphology, and apoptosis in mouse testicular tissue by inhibiting oxidative stress and promoting anti-apoptosis [19]. Moreover, GL extract alleviated Li_2_CO_3_-induced testicular damage in mice and protected the testes by restoring germ cell generation and differentiation [20]. Another study had also shown that a novel karaya gum micro-particle-loaded GL polysaccharide could prevent testicular inflammation and oxidation caused by cadmium as well as protect the histological characteristics of normal testicular tissue [21]. *Ganoderma lucidum* polysaccharide peptide (GLPP) is a mixture of polysaccharide peptides extracted from GL. Previous studies have shown that GLPP has various pharmacological activities including antioxidation, anti-inflammation, and regulating immune function [22,23,24,25,26]. These data indicate that GLPP may have a protective effect on the male reproductive system against damage caused by chemotherapy.

Spermatogenesis is a sophisticated biological process responsible for the development of spermatozoa from spermatogonial stem cells [27]. The process of male spermatogenesis includes three successive stages, namely proliferation and differentiation of spermatogonial stem cells (stage 1), spermatocyte meiosis (stage 2), and differentiation of haploid spermatids into sperm (spermiogenesis) (stage 3) [28,29]. Spermatogonial stem cells take about 35 days to develop into mature sperms [30,31], during which they are easily damaged by chemotherapy thus impairing final sperm formation [32,33,34]. Therefore, it is interesting to clarify the protective mechanism of GLPP on the spermatogenic process.

In this study, the protective effect of GLPP on the male reproductive system was explored through a chemotherapeutic drug cyclophosphamide (CP)-treated mouse model [35,36,37]. The experimental results showed that CP significantly damaged spermatogenesis in mice; however, GLPP could ameliorate the testicular damage caused by CP at three different stages of the spermatogenesis. Our study provides a proof of concept for the pharmacological effect of GLPP in preventing chemotherapy-induced male reproductive system damage, suggesting that GLPP may be developed into the preventive drugs or functional foods for adult patients with chemotherapy.

## 2. Materials and Methods

### 2.1. Chemicals

GLPP was isolated and purified by the National Engineering Research Center of Juncao Technology. The average molecular weight of GLPP was approximately 520 kDa. Cyclophosphamide (CP, purity > 95%) was purchased from Shanghai Macklin Biochemical Co., Ltd. (Shanghai, China). GLPP and CP were freshly dissolved in normal saline before administration.

### 2.2. Animals

Male ICR mice (8~10 weeks old, 28~30 g) were purchased from the Laboratory Animal Center of Peking University (Beijing, China). The mice were provided with food and water ad libitum and maintained in a temperature-controlled environment (25 ± 1 °C) in a 12/12 h light–dark cycle. All animal care protocols were approved by the Institutional Animal Care and Use Committee at the Peking University Health Science Center (Approved number: LA220354, 19 May 2020, Peking University, Beijing, China). The study was carried out according to ARRIVE guidelines (https://www.nc3rs.org.uk/arrive-guidelines (accessed on 23 September 2022)) and the National Research Council’s guide for the Care and Use of Laboratory Animals (https://grants.nih.gov/grants/olaw/guide-for-the-care-anduse-of-laboratory-animals.pdf (accessed on 23 September 2022)). 

### 2.3. Animal Modeling 

**Figure 1:**
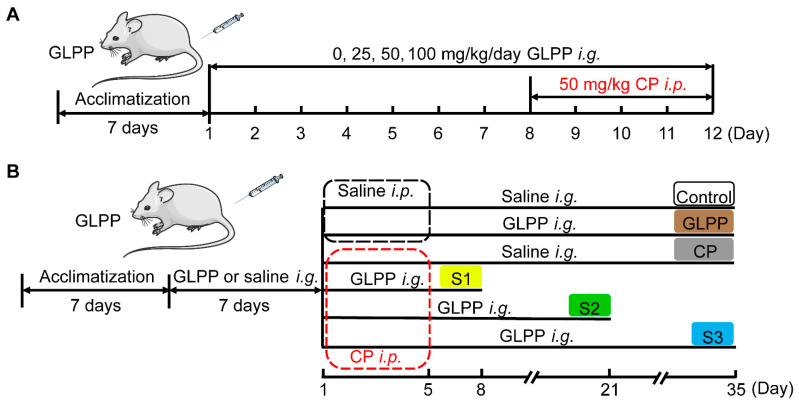
The schedule of the animal experiments.

To study the protective effect of GLPP on the male reproductive system from CP damage, mice were randomly divided into six groups after acclimatization for 1 week. The blank control group (Veh group) were gavaged with normal saline daily from day 1 to day 12. From the 8th day, saline (vehicle of CP) was injected intraperitoneally at 1 h after the daily gavage. The GLPP control group (GLPP) was gavaged with 100 mg/kg GLPP daily from day 1 to day 12. From the 8th day, saline (vehicle of CP) was injected intraperitoneally at 1 h after gavage daily. The CP group (CP) were gavaged daily with normal saline from day 1 to day 12. From the 8th day, CP (50 mg/kg) was injected intraperitoneally daily at 1 h after gavage. The low dose GLPP group (CP + 25 mg/kg GLPP), medium dose GLPP group (CP + 50 mg/kg GLPP), and high dose GLPP group (CP + 100 mg/kg GLPP) were gavaged with GLPP (25, 50, or 100 mg/kg) daily from day 1 to day 12. From the 8th day, CP (50 mg/kg) was injected intraperitoneally daily at 1 h after gavage. The schedule of the above experiment arrangement is shown in Figure 1A. At the end of experiments, the mice were anesthetized with isoflurane and tissue samples were obtained for bioanalysis, histopathological examination, and molecular biological analysis. 

To study the effect of GLPP on spermatogenic stages 1, 2, and 3, the modeling time was determined as previously described [28,38]. The specific group arrangements are shown in Figure 1B. In group I (Control), the mice were gavaged with saline daily from day 1 to day 35 after saline pretreatment for 7 days. From day 1 to day 5, saline was injected intraperitoneally 1 h after the daily gavage. In group II (GLPP), the mice were gavaged with 100 mg/kg GLPP daily from day 1 to day 35 after GLPP pretreatment for 7 days. From day 1 to day 5, saline was injected intraperitoneally 1 h after the daily gavage. In group III (CP), the mice were gavaged with saline daily from day 1 to day 35 after saline pretreatment for 7 days. From day 1 to day 5, CP (50 mg/kg) was injected intraperitoneally 1 h after the daily gavage. In group IV (Stage 1, S1) were gavaged with 100 mg/kg GLPP daily from day 1 to day 8 after GLPP pretreatment for 7 days. From day 1 to day 5, CP (50 mg/kg) was injected intraperitoneally 1 hour after the daily gavage. In group V (Stage 2, S2), the mice were gavaged with GLPP (100 mg/kg) daily from day 1 to day 21 after GLPP pretreatment for 7 days. From day 1 to day 5, CP (50 mg/kg) was injected intraperitoneally 1 h after the daily gavage. In group VI (Stage 3, S3), the mice were gavaged with GLPP (100 mg/kg) daily from day 1 to day 35 after GLPP pretreatment for 7 days. From day 1 to day 5, CP (50 mg/kg) injected intraperitoneally 1 h after the daily gavage. After the last dose, all mice were anesthetized with isoflurane for the evaluation of various parameters within 24 h.

### 2.4. Reproductive Organs Index 

The testes and epididymides were collected and weighed. The testicular and epididymal indexes were calculated by dividing the weight of the testes (mg) or epididymides (mg) by the body weight (g).

### 2.5. Evaluation of Sperm Parameters

The cauda epididymides were cut into pieces in saline and incubated at 37 °C for 10 min in a 5% CO_2_ incubator. Then, the sperm were gently filtered through a nylon gauze. A drop of the sperm suspension was assessed for sperm count and motility parameters by (CASA). For examining sperm morphology, a 20 μL sperm suspension was spread on a glass slide. Sperm morphology was then visualized by eosin staining. A total of 1000 sperm cells per sample were observed to identify any abnormalities. Sperm with shape abnormalities, such as banana, headless, without hook, amorphous, double-tailed, and fat head, were identified and counted.

### 2.6. Histopathological Assessment of Testicular Tissue 

The testicular tissue samples were prepared using the standard paraffin embedding method, then sectioned into 5 µm thick sections, and finally stained with hematoxylin and eosin (H&E) for morphological analysis. The spermatogenesis was graded from 1 to 10 using the Johnsen mean testicular biopsy score [39] as follows: Score 10, complete spermatogenesis and intact structure of tubules; score 9, many spermatozoa and slight disorganization of tubules; score 8, a small number of spermatozoa; score 7, no spermatozoa with many spermatids; score 6, no spermatozoa, only a few spermatids; score 5, no spermatozoa and spermatids, many spermatocytes, score 4, only some spermatocytes; score 3, only spermatogonial cells; score 2, only Sertoli cells with no germ cells; score 1, neither germ cells nor Sertoli cells. Finally, the final Johnsen score was assigned through dividing the total score by the number of assessed tubules.

### 2.7. Biochemical Analysis

Testicular tissues were collected from each group. The tissues were homogenized with cold saline to make 1% tissue homogenate and centrifuged at 12,000× *g* for 15 min at 4 °C, then the supernatant was collected. Superoxide dismutase (SOD), malonaldehyde (MDA), glutathione (GSH), and catalase (CAT) were measured to determine the degree of testicular injury using a colorimetric assay kit (Nanjing Jiancheng Bioengineering, Nanjing, China) according to the manufacturer’s instructions.

### 2.8. Assessment of Serum Hormones 

Blood was collected from the mouse retroorbital venous plexus using a capillary tube and centrifuged at 3000× *g* for 15 min at 4 °C to obtain serum. According to the kit manufacturer’s protocol, serum was kept for the analysis of the follicle-stimulating hormone (FSH) and testosterone (T) via an Enzyme-Linked Immunosorbent Assay (ELISA) (USCN Life Science, Wuhan, China).

### 2.9. Western Blot Analysis

The testis tissue was homogenized in RIPA lysis buffer, containing a protease inhibitor cocktail (Roche, Basel, Switzerland) and phosphatase inhibitor, on ice. The supernatant was collected after centrifugation. Protein was then quantified using a BCA protein assay kit (Thermo Scientific, Waltham, MA, USA). Equal amounts of protein were separated by SDS-PAGE and blotted onto polyvinylidene difluoride (PVDF) membranes (Amersham Biosciences, Boston, MA, USA). After being blocked with 5% skimmed milk for 2 h, the membranes were washed by TBST (20 mM Tris-HCl, 137 mM NaCl, and 0.1% Tween-20, pH 7.4) and incubated with the relevant primary antibody (Nrf2, A0674; HO-1, A1346; Keap-1, A1820; Bax, A0207; Bcl-2, A21592 and β-actin, AC004) for 12 h at 4 °C. Following incubation, the membranes were washed 3 times using TBST and probed with horseradish peroxidase-labeled secondary antibody (goat anti-mouse or anti-rabbit IgG) for 2 h at room temperature. The blots were then developed using a super-sensitive ECL luminescence reagent (Meilunbio, Dalian, China). The protein bands were visualized using a chemiluminescence detection system (Syngene, GeneGnome XRQ, Cambridge, Cambridgeshire, UK) and analyzed using ImageJ (ij153-win-java8) software (NIH, Bethesda, MD, USA).

### 2.10. Statistical Analysis

Statistical analyses were carried out using GraphPad Prism 8. All data were presented as mean ± SEM. The difference between the two groups was analyzed by Student’s *t*-test. Multiple group comparisons were analyzed using a one-way analysis of variance (ANOVA) with Tukey’s correction. A *p*-value < 0.05 was considered statistically significant.

## 3. Results

### 3.1. GLPP Protected Male Reproductive System against CP Injury

**Figure 2:**
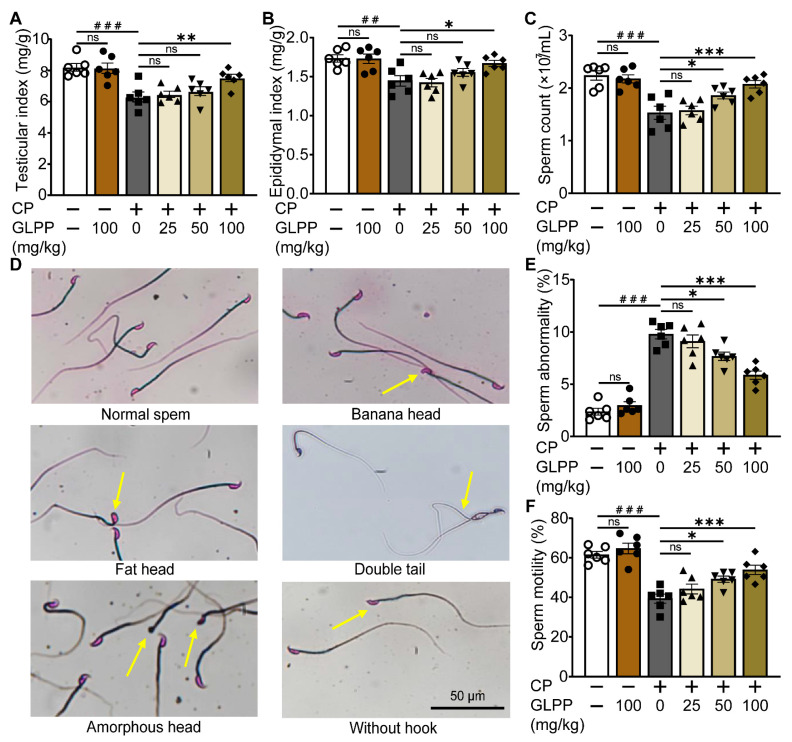
Effect of GLPP on CP-induced male reproductive injury in mice. (**A**) Testicular index. (**B**) Epididymal index. (**C**) Sperm count. (**D**) The representative photographs of sperm morphology from epididymis. The yellow arrows indicate the sperm with abnormal morphology (**E**) Sperm abnormality. (**F**) Sperm motility. Data are presented as mean ± SEM (n = 6). ## *p* < 0.01 and ### *p* < 0.001 vs. Ctr; * *p* < 0.05, ** *p* < 0.01 and *** *p* < 0.001 vs. CP; ns, no significance.

The mouse model with CP-induced male reproductive injury was established as scheduled in Figure 1A. Compared with the control group, CP significantly lowered the testicular and epididymal indexes (Figure 2A,B) and sperm counts (Figure 2C) in the mice. Meanwhile, the percentage of sperm with abnormal morphology was significantly increased in the mice with CP-induced injury (Figure 2D,E). The motility of the sperm was also reduced by CP (Figure 2F). Interestingly, these CP-induced abnormalities were significantly restored by GLPP in a dose-dependent manner, which indicates that GLPP can prevent CP-induced male reproductive injury.

### 3.2. GLPP Ameliorated Testicular Histological Injury Induced by CP

**Figure 3:**
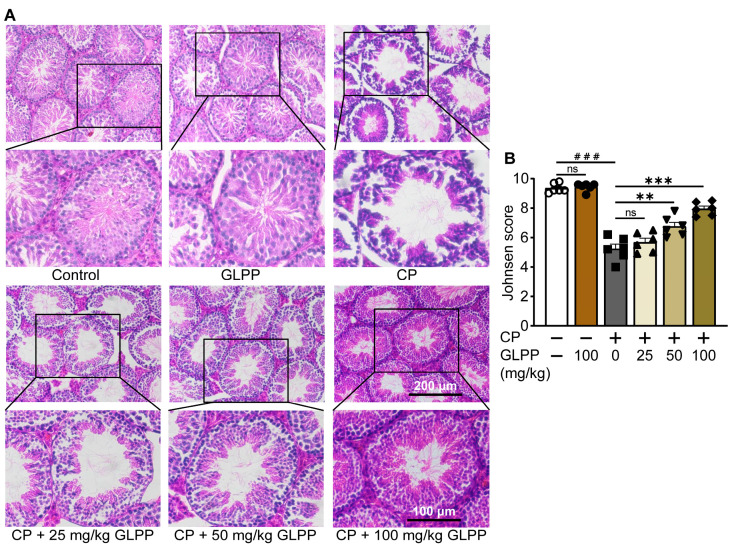
Histopathology of testis. (**A**) Representative images of testicular H&E staining. (**B**) Johnsen score of the seminiferous tubules. Data are presented as mean ± SEM (n = 6). ### *p* < 0.001 vs. Ctr; ** *p* < 0.01 and *** *p* < 0.001 vs. CP; ns, no significance.

The mouse testes in the control and GLPP groups exhibited similar testicular tissue morphology, with a majority of the seminiferous epithelium containing different stages of spermatogenic cells in well-stratified sequences (Figure 3A). However, in the CP-treated mice, the cavity of the seminiferous tubules was enlarged, while the seminiferous epithelium was either thin or incomplete, and the various spermatogenic cells were decreased. Most tubules had very few spermatozoa in the lumen, suggesting that the spermatogenic function was damaged by CP. After administration of GLPP, the lumen of the seminiferous tubules was significantly improved in a dose-dependent manner. The increase in the number and layers of spermatogenic cells in the lumen suggested that GLPP alleviated CP-induced changes in the testis, particularly under the administration of a high dose of GLPP. Furthermore, CP decreased the Johnsen score of the testicular sections, which indicates testicular injury; however, 50 and 100 mg/kg GLPP was able to reverse the score (Figure 3B).

### 3.3. GLPP Alleviated CP-Induced Spermatogenic Dysfunction at Stages of S1–S3

**Figure 4:**
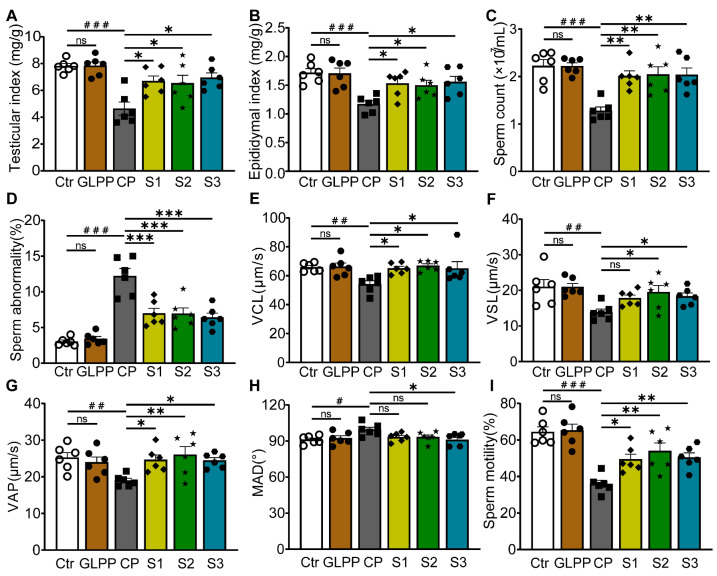
Organ indexes and sperm parameters. (**A**) Testicular index. (**B**) Epididymal index. (**C**) Sperm count. (**D**) Sperm abnormality. (**E**–**H**) Sperm kinematic parameters. (**I**) Sperm motility. Data are presented as mean ± SEM (n = 6). # *p* < 0.05, ## *p* < 0.01 and ### *p* < 0.001 vs. Ctr; * *p* < 0.05, ** *p* < 0.01 and *** *p* < 0.001 vs. CP; ns, no significance.

The process of male spermatogenesis includes three stages, namely proliferation and differentiation of spermatogonial stem cells (Stage1, S1), spermatocyte meiosis (Stage2, S2), and spermatogenesis (Stage3, S3). After the mice were given high doses of GLPP and CP, the samples were taken at different times to determine which stage of spermatogenic damage GLPP mainly protected against (Figure 1B). It was found that CP significantly lowered the testicular and epididymal indexes, sperm counts, sperm motility, and sperm kinematic parameters (including curve–linear velocity (VCL), straight-line velocity (VSL), and average path velocity (VAP)), and significantly increased sperm abnormality and average motion degree (MAD) compared with the control group. Interestingly, in the S1–3 groups, GLPP significantly reversed the testicular and epididymal indexes and improved the sperm kinematic parameters changed by CP, resulting in levels that were close to those of the control group (Figure 4A–I).

### 3.4. GLPP Ameliorated Tissue Injuries of Testis

**Figure 5:**
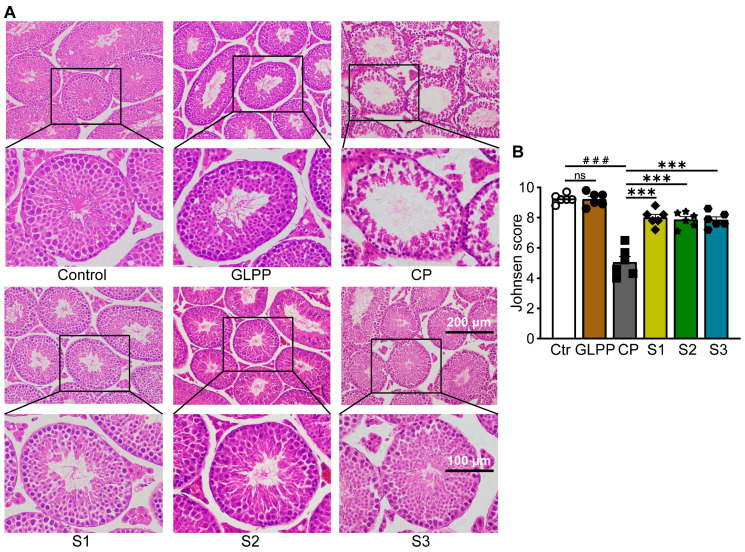
Tissue morphology of the testis. (**A**) Representative images of testicular H&E staining. (**B**) Johnsen’s score of the seminiferous tubules. Data were presented as mean ± SEM (n = 6). ### *p* < 0.001 vs. Ctr; *** *p* < 0.001 vs. CP; ns, no significance.

After CP treatment, the number of spermatogenic cells (spermatocytes, spermatozoa, and spermatogonia) decreased significantly, resulting in dilation of the seminiferous tubules (Figure 5A). In the S1–3 groups, GLPP significantly increased the layers of spermatogenic cells in the lumen of seminiferous tubules as well as the number of sperm in CP-treated mice to near normal levels. In particular, the number of mature spermatogenic cells entering the lumen was significantly increased, indicating a more significant improvement in meiotic and post-meiotic cells. The Johnsen score of the testicular tissue sections was significantly increased by GLPP as shown in Figure 5B.

### 3.5. GLPP Enhanced Testicular Antioxidant Capacity

**Figure 6:**
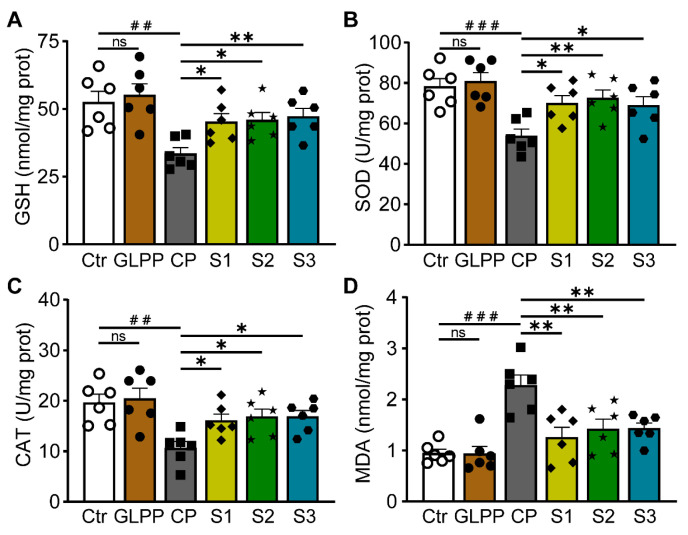
GSH, SOD, CAT activity and MDA content in the testicular tissue. (**A**) GSH activity. (**B**) SOD activity. (**C**) CAT activity. (**D**) MDA content. Data were presented as mean ± SEM (n = 6). ## *p* < 0.01 and ### *p* < 0.001 vs. Ctr; * *p* < 0.05 and ** *p* < 0.01 vs. CP; ns, no significance.

GSH, SOD, CAT, and MDA are four important oxidative stress indicators. GSH can effectively scavenge free radicals [40]. SOD is a widespread free radical scavenger [41]. CAT plays an important role in scavenging free radicals [42]. MDA reflects the degree of peroxidation in the body [43]. The protective effect of GLPP on CP-induced oxidative stress was evaluated by measuring the levels and activities of GSH, SOD, CAT, and MDA in the testis tissues. Compared with the control group, CP significantly decreased the activities of GSH (Figure 6A), SOD (Figure 6B), and CAT (Figure 6C), and increased MDA content (Figure 6D) in the testes. In the S1–3 groups, GLPP significantly raised the activity levels of GSH, SOD, and CAT, and decreased the content of MDA in CP-treated mice, indicating that GLPP effectively enhances the antioxidant capacity of spermatogenic cells at different stages in mouse testes, thereby alleviating CP-induced oxidative stress damage.

### 3.6. GLPP Restored Sex Hormone Levels

**Figure 7:**
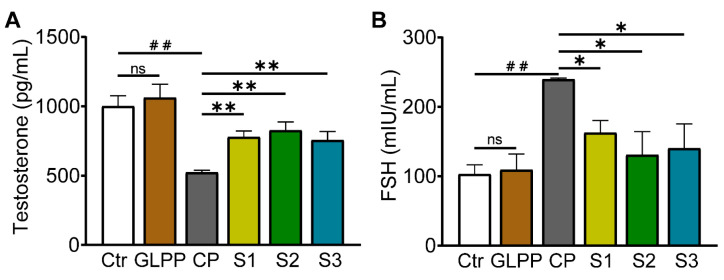
Testosterone and follicle-stimulating hormone (FSH) concentrations in mouse serum. (**A**) Testosterone concentration. (**B**) FSH concentration. Data were presented as mean ± SEM (n = 3). ## *p* < 0.01; * *p* < 0.05 and ** *p* < 0.01 vs. CP; ns, no significance.

Testosterone is the most important sex hormone in males and plays a vital role in testicular development, spermatogenesis, and the maintenance of normal virilization [44]. The follicle-stimulating hormone (FSH) induces the proliferation of Sertoli cells in the testes, thereby initiating and maintaining normal spermatogenesis [45]. Therefore, measurements of the serum FSH and testosterone concentrations without exogenous hormone intervention can be used as important indicators to evaluate spermatogenic function. Compared with the control group, CP significantly decreased testosterone levels (Figure 7A) and increased FSH level (Figure 7B). In the S1–3 group, GLPP significantly increased testosterone levels and decreased FSH levels in CP-treated mice, suggesting that GLPP can improve the reproductive hormone endocrine disorders caused by CP and have a pharmacological effect on sperm maturation and spermatogenesis.

### 3.7. GLPP Ameliorated Testicular Injury by Activating the Keap1/Nrf2/HO-1 Signaling Pathway

**Figure 8:**
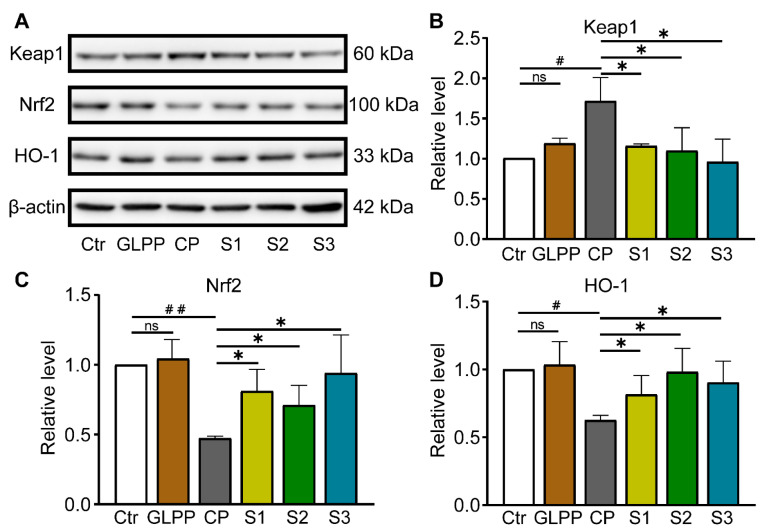
Keap-1, Nrf2, and HO-1 protein expressions in testicular tissue. (**A**) Representative Western blots of Keap-1, Nrf2, HO-1 and β-actin. Relative levels of Keap1 (**B**), Nrf2 (**C**) and HO-1 (**D**) quantified by the band intensity. Data were presented as mean ± SEM (n = 4). # *p* < 0.05 and ## *p* < 0.01 vs. Ctr; * *p* < 0.05 vs. CP; ns, no significance.

The nuclear factor-erythroid 2–kelch-like ECH-associated protein 1(Nrf-2/Keap-1)/Heme-oxygenase1 (HO-1) signaling pathway plays a vital role in shielding cells from intracellular oxidative stress [46,47]. Given the importance of Keap1/Nrf2/HO-1 signaling in oxidative stress, we were determined to examine GLPP function in the mediation of this pathway. Western blot analyses of the Keap1, Nrf2, and HO-1 protein expression levels are shown in Figure 8A. As compared to the control group, CP increased Keap1 protein expression (Figure 8B), while greatly lowering the Nrf2 and HO-1 protein expression levels (Figure 8C,D) in the testes. GLPP significantly increased the CP-reduced Nrf2 and HO-1 expression levels. At the same time, GLPP remarkably inhibited the CP-increased Keap1 expression. These data suggest that GLPP protects the mouse testes from CP-induced oxidative stress by activating the Keap1/Nrf2/HO-1 signaling pathway.

### 3.8. GLPP Had Anti-Apoptotic Effect via Regulating Bax/Bcl-2 Pathway

**Figure 9:**
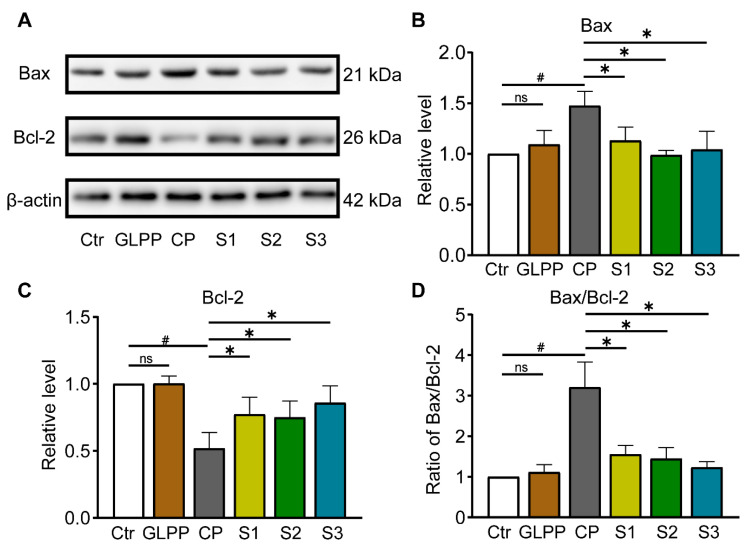
Protein expressions involved in Bax/Bcl-2 signaling pathway in the testicular tissue. (**A**) Representative Western blots of Bax, Bcl-2, and β-actin. The relative protein expression levels of Bax (**B**), Bcl-2 (**C**) and Bax/Bcl-2 ratio (**D**). Data are presented as mean ± SEM (n = 4). # *p* < 0.05 vs. Ctr; * *p* < 0.05 vs. CP; ns, no significance.

By Western blot analysis (Figure 9A), we additionally assessed the expression levels of Bax and Bcl-2. In comparison to control group, Bax expression was higher (Figure 9B), while Bcl-2 expression was lower (Figure 9C), and the ratio of Bax/Bcl-2 (Figure 9D) were increased in testis tissue of CP-treated mice, in which GLPP was able to significantly reverse the effect of CP, thereby protecting the germ cells from CP-induced apoptosis in the three stages of spermatogenesis.

## 4. Discussion

Recent studies have shown that male patients undergoing chemotherapy suffer from sperm quality problems, including oligospermia, which usually leads to male infertility [48,49]. Therefore, there is an urgent need to develop safe and effective drugs to prevent and treat chemotherapy-induced male infertility. It has been found that some antioxidant compounds derived from various animals and plants play certain protective roles in the male reproductive system [50,51,52]. The motivation of this study was to determine whether GLPP could ameliorate the male reproductive damage caused by chemotherapeutic drugs in a mouse model. Our experimental results showed that GLPP had a significant protective effect on germ cells at different spermatogenic stages by reducing oxidative stress and cell apoptosis.

Previous studies have shown that chemotherapy-induced intracellular oxidative stress is a critical factor leading to testicular toxicity [53]. CP, an alkylating agent commonly used in tumor therapy, is metabolized in vivo through the cytochrome P450 system expressed in the liver and testes to produce toxic electrophiles such as aldophosphamide mustard and acrolein [54,55,56]. It has been reported that CP can disrupt the proliferation and differentiation of spermatogonial stem cells, induce apoptosis of spermatocytes, destroy the genetic material of germ cells, and reduce spermatogonia [49,57,58,59]. CP is commonly used as tool (drug) in male animal models to study oligozoospermia [60,61]. Our study found that CP decreased the organ coefficients of the testis and epididymis as well as the count and viability of sperm in the model mice, in which the sperm abnormality rates were elevated. The histopathological examination revealed the disorganization of spermatogonial tubules in the testis and a decrease in the number of spermatozoa in the lumen of the tubules of the CP-treated mice, suggesting that CP damages testicular tissue and spermatogenesis.

The testis is highly susceptible to oxidative stress due to the abundance of ROS-generating systems (xanthine oxidase, nicotinamide adenine dinucleotide phosphate (NADPH) oxidase, and mitochondrial electron transport chain) and high concentrations of polyunsaturated fatty acids (PUFA) [62,63,64,65]. Thus, the antioxidant system plays a crucial role in protecting germ cells against oxidative damage [66,67]. Antioxidants include synthetic antioxidants and natural antioxidants. Natural antioxidant substances have been given a lot of attention because of their high efficiency, low toxicity, and wide range of sources [68].

GLPP is a group of polysaccharide peptides extracted and purified from GL. It was found that the GLPP significantly reduced the oxidative stress and apoptosis of myocardial cells in vitro [69]. In addition, supplementation with GLPP in a cryopreservation medium improved the antioxidant enzyme activities to protect against ROS/OS-induced cryodamage in the post-thawed sperm [70]. GLPP could play a good ameliorative role, through antioxidants, in the severe damage caused by lithium carbonate related to oxidative stress in the male reproductive system [20]. Our experimental results proved that, compared with the CP model group, GLPP (50 and 100 mg/kg) maintained the testis and epididymis indexes, improved sperm parameters, decreased sperm abnormality rate, and attenuated the pathologic damage of testicular tissues to varying degrees in the CP-treated mice, which indicates that GLPP could ameliorate the CP-induced spermatogenic injury. In addition, compared with the control mice, there was no significant difference in the experimental indexes of mice treated with the high-dose GLPP, indicating that GLPP did not damage the spermatogenic function of mice.

Based on the above experimental results, we performed experiments on the spermatogenesis cycle and the timing of the spermatogenesis stages. In order to retrospectively study which stage of spermatogenesis GLPP exerts a better protective effect after the application of CP, we took samples at specific time points after the administration of GLPP to observe the sperm indicators. The results showed that after GLPP treatment, the sperm count, motility, kinematic parameters, and sex hormone levels of the S1–S3 stages in the CP-treated mice were significantly improved.

The CP metabolites not only disrupt the oxidative balance and antioxidant system in the testicular tissue, but they also reduced antioxidant enzyme activity, which resulted in a large accumulation of oxygen free radicals and their peroxides in the organism thus causing oxidative stress injury in testicular tissues [71,72,73]. The joint detection of the Nrf2, HO-1, SOD, GSH, CAT activities and the MDA content in the testicular tissues can accurately reflect the antioxidant capacity of the tissues and the degree of oxidative stress damage [74,75]. Our study showed that intraperitoneal injection of CP resulted in a dramatic increase in the testicular oxidative stress indices, as evidenced by the high MDA levels and the corresponding inhibition of testicular antioxidant enzyme capacities (SOD, GSH, and CAT), and a significant decrease in the protein content of Nrf2 and its downstream HO-1 in testicular tissues. GLPP decreased MDA in the testicular tissue of CP-treated mice at all three spermatogenic stages, while increasing testicular GSH, SOD, and CAT activities, and significantly increasing Nrf2 and HO-1 expressions in testicular tissue, which significantly enhanced the physiological antioxidant responses of the testicular tissue.

Oxidative stress not only disrupts intracellular homeostasis, but also leads to the apoptosis of spermatogenic cells [76,77]. Bcl-2 and Bax are pivotal regulatory proteins for the mitochondria-mediated apoptosis process [78,79]. The ratio of Bax/Bcl-2 expression in testicular tissues can reflect whether the cells tend to apoptosis or survival after CP treatment. In this study, CP significantly increased the Bax/Bcl-2 ratio in the testicular tissue of the mice, in which GLPP significantly reversed the Bax/Bcl-2 ratio. Also, GLPP significantly alleviated the damage caused by CP in the testicular tissue. Therefore, reducing apoptosis may be one of the mechanisms through which GLPP protects testicular tissue structure and function from damages.

## 5. Conclusions

This study demonstrated that GLPP can dose-dependently alleviate the CP-induced testicular injury. By regulating the Keap1/Nrf2/HO-1-mediated oxidative stress pathway and Bax/Bcl-2-mediated apoptosis pathway, GLPP ameliorates the damage to male reproductive function induced by CP and has a protective effect on the spermatogenic cells at different stages. Our study suggests that GLPP might be developed as a candidate drug to prevent and treat chemotherapy-induced male reproductive impairment.

## Data Availability

Data are contained within the article.
